# Supplementary figures and images for: Effect of Pd/ZnO Morphology on Surface Acoustic Wave Sensor Response
Source: Nanomaterials (Basel). 2021 Oct 2;11(10):2598. doi: 10.3390/nano11102598 (PMC8538218; doi:10.3390/nano11102598)

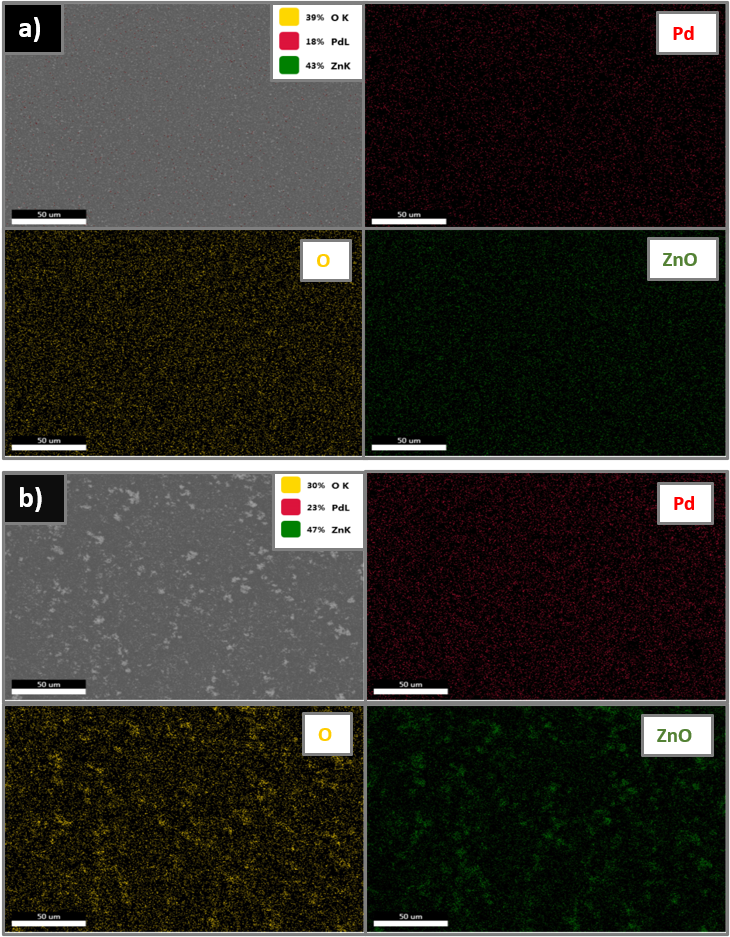

Supplement: Supplementary file 1 [file nanomaterials-11-02598-s001.zip › nanomaterials-1392193-supplementary.tif]
